# Preclinical validation of 3-phosphoinositide-dependent protein kinase 1 inhibition in pancreatic cancer

**DOI:** 10.1186/s13046-019-1191-2

**Published:** 2019-05-14

**Authors:** Aikaterini Emmanouilidi, Chanse A. Fyffe, Riccardo Ferro, Charlotte E. Edling, Emily Capone, Simona Sestito, Simona Rapposelli, Rossano Lattanzio, Stefano Iacobelli, Gianluca Sala, Tania Maffucci, Marco Falasca

**Affiliations:** 1 Metabolic Signalling Group, School of Pharmacy and Biomedical Sciences, Curtin Health Innovation Research Institute, Curtin University, Perth, Western Australia 6102 Australia; 2 Queen Mary University of London, Barts and The London School of Medicine and Dentistry, Blizard Institute, Centre for Cell Biology and Cutaneous Research, E1 2AT, London, UK; 3 Dipartimento di Scienze Mediche, Orali e Biotecnologiche, University G. d’Annunzio di Chieti-Pescara, Centro Studi sull Invecchiamento, CeSI-MeT, 66100 Chieti, Italy; 4 Department of Pharmacy, University of Pisa, Via Bonanno, 6, 56126 Pisa, Italy; 5 MediaPharma Srl, Via della Colonnetta, 50/A, 66100 Chieti, Italy

**Keywords:** Pancreatic ductal adenocarcinoma, Signal transduction, Targeted therapy, Phosphoinositide 3-kinase, 3-phosphoinositide-dependent protein kinase 1, Serum/glucocorticoid regulated kinase family member 3

## Abstract

**Background:**

The very aggressive nature and low survival rate of pancreatic ductal adenocarcinoma (PDAC) dictates the necessity to find novel efficacious therapies. Recent evidence suggests that phosphoinositide 3-kinase (PI3K) and 3-phosphoinositide-dependent protein kinase 1 (PDK1) are key effectors of oncogenic KRAS in PDAC. Herein, we report the role and mechanism of action of PDK1, a protein kinase of the AGC family, in PDAC.

**Methods:**

PDAC cell lines were treated with selective PDK1 inhibitors or transfected with specific PDK1-targeting siRNAs. In vitro and in vivo assays were performed to investigate the functional role of PDK1 in PDAC. Specifically, anchorage-dependent and anchorage-independent growth was assessed in PDAC cells upon inhibition or downregulation of PDK1. Detailed investigation of the effect of PDK1 inhibition/downregulation on specific signalling pathways was also performed by Western blotting analysis. A xenograft tumour mouse model was used to determine the effect of pharmacological inhibition of PDK1 on PDAC cells growth in vivo.

**Results:**

Treatment with specific inhibitors of PDK1 impaired anchorage-dependent and anchorage-independent growth of pancreatic cancer cell lines, as well as pancreatic tumour growth in a xenograft model. Mechanistically, inhibition or downregulation of PDK1 resulted in reduced activation of the serum/glucocorticoid regulated kinase family member 3 and subsequent reduced phosphorylation of its target N-Myc downstream regulated 1. Additionally, we found that combination of sub-optimal concentrations of inhibitors selective for PDK1 and the class IB PI3K isoform p110γ inhibits pancreatic cancer cell growth and colonies formation more potently than each single treatment.

**Conclusions:**

Our data indicate that PDK1 is a suitable target for therapeutic intervention in PDAC and support the clinical development of PDK1 inhibitors for PDAC.

**Electronic supplementary material:**

The online version of this article (10.1186/s13046-019-1191-2) contains supplementary material, which is available to authorized users.

## Background

Pancreatic ductal adenocarcinoma (PDAC) is amongst the five most deadly human malignancies, having a 5-year relative survival rate of ~8% [1]. PDAC is associated with high-frequency somatic mutations in a subset of genes, most frequently in the gene encoding the small GTPase KRAS, which is mutated in the majority of human PDAC (> 95%) [2]. The genes encoding the tumour suppressor p53, SMAD4 and p16^Ink4A^ are also frequently mutated in PDAC with many other accessory mutations also being observed at varied frequency [3].

Since their discovery, phosphoinositide 3-kinases (PI3Ks) have been established as major signalling molecules implicated in different cellular functions such as glucose metabolism, cellular proliferation, cellular survival and angiogenesis. Abnormal PI3K signalling has been estimated to occur in as many as 50% of all human malignancies and this pathway is a well-established target for anti-cancer therapies [4]. The PI3K family comprises of eight mammalian isoforms grouped into three classes. Class IA consists of a catalytic subunit and a regulatory subunit. The catalytic subunits include p110α, p110β, or p110δ, while the regulatory subunits consist of p85α, p85β or p55γ. Class IB consists of only one catalytic subunit, p110γ, and two regulatory subunits, p87 and p101. We previously reported that p110γ is overexpressed in human PDAC and plays a key role in pancreatic cancer cell proliferation [5]. These data were confirmed by a recent study also reporting increased levels of p110γ in human pancreatic cancer tissues as well as its role in regulation of pancreatic cancer cell growth [6]. Similarly, p110γ overexpression was detected in human hepatocellular carcinoma (HCC) where the enzyme was also shown to be a key regulator of cellular proliferation [7].

In response to receptor tyrosine kinases or G-protein coupled receptors activation, class I PI3Ks catalyse the phosphorylation of the 3′ position of the inositol ring of phosphatidylinositol 4,5-bisphosphate [PtdIns(4,5) *P*_*2*_] which leads to the synthesis of the membrane bound phospholipid phosphatidylinositol 3,4,5-trisphosphate [PtdIns(3,4,5) *P*_*3*_] [8]. PtdIns(3,4,5) *P*_*3*_ then acts as a second messenger and regulates both plasma membrane translocation and activation of several proteins. The tumour suppressor phosphatase and tensin homolog (PTEN) inhibits this signalling pathway by dephosphorylating PtdIns(3,4,5) *P*_*3*_ back to PtdIns(4,5) *P*_*2*_. The best characterized PI3K/PtdIns(3,4,5) *P*_*3*_ downstream effector is the Serine/Threonine-specific protein kinase B (PKB)/Akt that binds to PtdIns(3,4,5) *P*_*3*_ via its pleckstrin homology (PH) domain. Once at the plasma membrane, Akt is phosphorylated at its residue Thr308 by the 3-phosphoinositide-dependent protein kinase 1 (PDK1), which itself associates to the membrane via PH domain-dependent binding to PtdIns(3,4,5) *P*_*3*_, and at its residue Ser473 by other kinases, including the complex 2 of mechanistic target of rapamycin. PDK1 belongs to the family of AGC kinases and was first discovered in 1997 for its ability to phosphorylate Akt at Thr308. Although the contribution of PI3K and PI3K-dependent pathways to cancer development and progression has been well established for many years, the first major indication that PDK1 itself might be a viable target in cancer only appeared in 2005 when Bayascas et al generated transgenic hypomorphic PDK1 mice [9]. When these mice were crossed with tumourigenic heterozygous PTEN^+/−^ mice, the prevalence of tumour development was reduced in mice with deficient PDK1 levels, confirming the importance of PDK1 in tumour development driven by loss of PTEN [9]. Further evidence of a specific role for PDK1 in cancer is provided by the observation that increased copy number of *PDPK1*, the gene encoding for PDK1, is frequently observed in different cancer types [10–12]. Evidence is also emerging indicating that PDK1 inhibition can impact on several cellular functions associated with cancer progression, such as reduced invasion on Matrigel of breast, prostate and melanoma cancer cell lines [13–15]. Similarly, stable downregulation of PDK1 inhibited migration of the breast cancer cell line MDA-MB-231 and metastasis formation upon implantation of cells in immunodeficient mice [16]. PDK1 has also been shown to be implicated in ovarian cancer aggressiveness via a short form of the Ron receptor tyrosine kinase [17], and COLL11A1 [18], whereas its pharmacological inhibition had been previously found to be able to enhance the effect of chemotherapeutic drugs in the ovarian cancer cell line SKOV-3 cells [19]. Furthermore, PDK1 mRNA is correlated with poor survival rates in untreated HCC patients and is the most prominent factor in the time to recurrence prediction, post-operatively [20].

Whilst PDK1 is most commonly associated with Akt signalling, it has become increasingly evident that the role of PDK1 in cancer is not limited to Akt activation. PDK1 can phosphorylate and activate at least 23 AGC kinases including S6 Kinase, protein kinase C and serum-and glucocorticoid-induced protein kinase (SGK) [21]. The diversity of substrates that can be activated by PDK1 are of high relevance in cancer signalling. For instance, the PDK1 substrate serum/glucocorticoid regulated kinase family member 3 (SGK3) is frequently overexpressed in HCC and its downregulation reduced both colonies formation and tumour formation in nude mice [22]. In a subset of breast cancer cell lines, hyperactivation of PI3K pathways was reported to be independent from Akt activation and it was shown that tumourigenicity of the cells, as assessed by anchorage-independent growth, was dependent on PDK1 and SGK3 [23]. This provided the first evidence to suggest that PDK1 can represent an Akt-independent molecular target in human malignancies. It is now well established that PI3K/PDK1-dependent, Akt-independent signalling pathways can contribute to tumourigenesis [24, 25].

A previous study reported that deletion of *PDPK1* inhibits KRas^G12D^- driven PDAC development in a transgenic mouse model [26], revealing a key role for PDK1 in PDAC initiation. Whether pharmacological inhibition of the enzyme can inhibit PDAC progression remains to be established. Here we determined the effect of selective PDK1 inhibitors on PDAC growth in vitro and in vivo. This study identified PDK1 as a novel potential target to develop new treatment strategies in pancreatic cancer.

## Methods

### Cell culture and transfection

HPAF-II, AsPC-1, CFPAC-1 and PANC-1 cells were obtained from ATCC and grown in complete growth media (Eagle’s Minimum Essential Medium, RPMI-1640 Medium, Iscove’s Modified Dulbecco’s Medium and Dulbecco’s Modified Eagle Medium, respectively) supplemented with 10% FBS (Bovogen Biologicals) and 1X Penicillin-Streptomycin-Glutamine (HyClone) at 37 °C in a 5% CO_2_ atmosphere. HPDE cells were kindly provided by Prof H. Kocher (Queen Mary University of London) and were cultured in keratinocyte serum-free medium supplemented with epidermal growth factor (EGF) and bovine pituitary extract (Life Technologies, Inc.). hTERT-HPNE cells were obtained from ATCC and cultured in 75% DMEM without glucose supplemented with 25% Medium M3 Base (INCELL Corporation LLC), 5% FBS, 10 ng/ml human recombinant EGF, 5.5 mM D-glucose and 750 ng/ml puromycin. For serum starvation, cells were seeded in a 6-well plate at a density of 3.5 × 10^6^ cells/well and were serum starved for 24 h. After that, cells were stimulated with media containing 10% FBS for 1 h in the presence or absence of the indicated inhibitors.

Downregulation of PDK1 was obtained using the following siRNAs from Dharmacon: Sequence 1 ON-TARGETplus Standard GACCAGAGGCCAAGAAUUUUU; Sequence 2 ON-TARGETplus Standard (A4) CAAGAGACCUCGUGGAGAAUU. Downregulation of SGK3 was obtained using the following siRNAs from Qiagen: Gene Solution siRNA SI00101003 (SGKL 3) and Gene Solution siRNA SI00287588 (SGKL 6). Cells were transfected using 75 nM of siRNAs and DharmaFECT 1 and DharmaFECT 2 transfection reagents (Dharmacon) according to manufacturer’s instructions.

### Cell viability assay

Effect of the drugs on anchorage-dependent growth was assessed by trypan blue exclusion assay. Briefly, cells were seeded in 12-well plates at a density of 5 × 10^4^ cells/well and treated with different concentrations of drugs for 72 h. Cells were then trypsinized, complete media was added and 10 μl of cell suspension was mixed with trypan blue dye [1]. The mixture was loaded on a Neubauer chamber and the number of viable cells per mL was calculated as (number of viable cells / 4) × 10^4^, corrected for the dilution factor.

### Anchorage-independent growth – soft agar assay

In order to assess the long-term effect of the drugs and the PDK1/SGK3 downregulation on the ability of cells to form 3D colonies (tumourigenicity), anchorage-independent growth assays were performed. Six-well plates were coated with a mixture of 1% noble agar: 2XRPMI [1:1(v/v)] (bottom layer). Once the first layer had solidified, a second layer was added, comprising of 0.6% noble agar: 2XRPMI [1:1(v/v)] containing 10,000 cells and supplemented with the required inhibitor or corresponding vehicle.

Alternatively, 10,000 cells that had been transfected with siRNAs were plated. After the second layer had solidified, 1x RPMI was added and plates were kept in a humidified incubator, at 37 °C in a 5% CO_2_ atmosphere. After 5 weeks incubation, colonies were fixed and stained with Crystal Violet (0.05%), visualized with ChemiDoc XRS+ System (Bio-Rad) and quantified with ImageJ software.

### Cell lysis and Western blotting analysis

Cells were lysed using cold radioimmunoprecipitation assay buffer (150 mM sodium chloride, 1.0% NP-40 or Triton X-100, 0.5% sodium deoxycholate, 0.1% sodium dodecyl sulfate, 50 mM Tris HCl, pH 8.0) supplemented with 1X Protease/Phosphatase Inhibitor Cocktail (100X stock, Cell Signaling Technology). After sonication at 4 °C, lysates were centrifuged at 10,000 g for 10 mins at 4 °C. Supernatants were transferred to a 1.5 ml tube and protein concentrations were determined using the Direct Detect Assay-Free cards and the Direct Detect Spectrometer (Merck Millipore, Darmstadt, Germany). Samples (35 μg/lane) were separated by SDS-PAGE and transferred to nitrocellulose membranes. Membranes were incubated in TBS containing Tween-20 (0.05% v/v) and supplemented with 3% bovine serum albumin (TBST -BSA) at room temperature (RT) for 1 h followed by overnight incubation with primary antibodies at 4 °C. The following day, membranes were washed with TBST at RT (3 × 10 mins), and incubated for 1 h at RT with the appropriate secondary antibody (1:20,000). After three washes in TBST and one wash in TBS, membranes were incubated with Clarity Western ECL Blotting Substrates (Bio-Rad) and images were acquired using a ChemiDoc XRS+ System (Bio-Rad). Primary antibodies used were: pFoxO1 (Thr24)/FoxO3a (Thr32) (#9464), pAkt (Thr308) (#4056), pSGK3 (Thr320) (#5642), pNDRG1 (Thr346) (#3217), Akt (#9272), SGK3 (#8156), PDK1 (#3062), NDRG1 (#5196), GAPDH (#5174). All antibodies were purchased from Cell Signaling Technology. Antibodies were diluted in TBST-BSA (1:1000, apart from pSGK3, 1:500).

### In vivo experiments

Athymic CD-1 nu/nu mice (5–7 weeks old) were purchased from Charles River Laboratories (Calco, LC, Italy) and maintained under specific pathogen-free conditions with food and water provided ad libitum*.* The animals’ health status was monitored daily. Procedures involving animals and their care were established according to the institutional guidelines in compliance with national and international policies (Autorizzazione N 484/2016-PR Ministero della Salute). HPAF-II (3.5 × 10^6^) cells were injected subcutaneously into the right flank of mice. When xenografts became palpable, tumour-bearing mice were divided into two groups (*n* = 7) with mice receiving MP7 (75 mg/kg) or vehicle by oral gavage (5 days/week) for 3 weeks. For this purpose, MP7 was administered as a suspension in 0.5% methylcellulose + 0.4% tween 80. Tumour volumes were monitored every week using a caliper and volumes were calculated using the following formula: tumour volume = (length* width^2^)/2. The PDK1 inhibitor MP7 was synthesized as previously reported [27] and characterized as described in Additional file 1. For in vivo xenograft curves, *p* values were determined by Student’s t-test and considered significant at *p* < 0.05. All statistical analysis was performed with GraphPad Prism 5.0 software.

## Results

### PDK1 inhibition reduces pancreatic cancer cell growth in vitro and in vivo

**Fig. 1:**
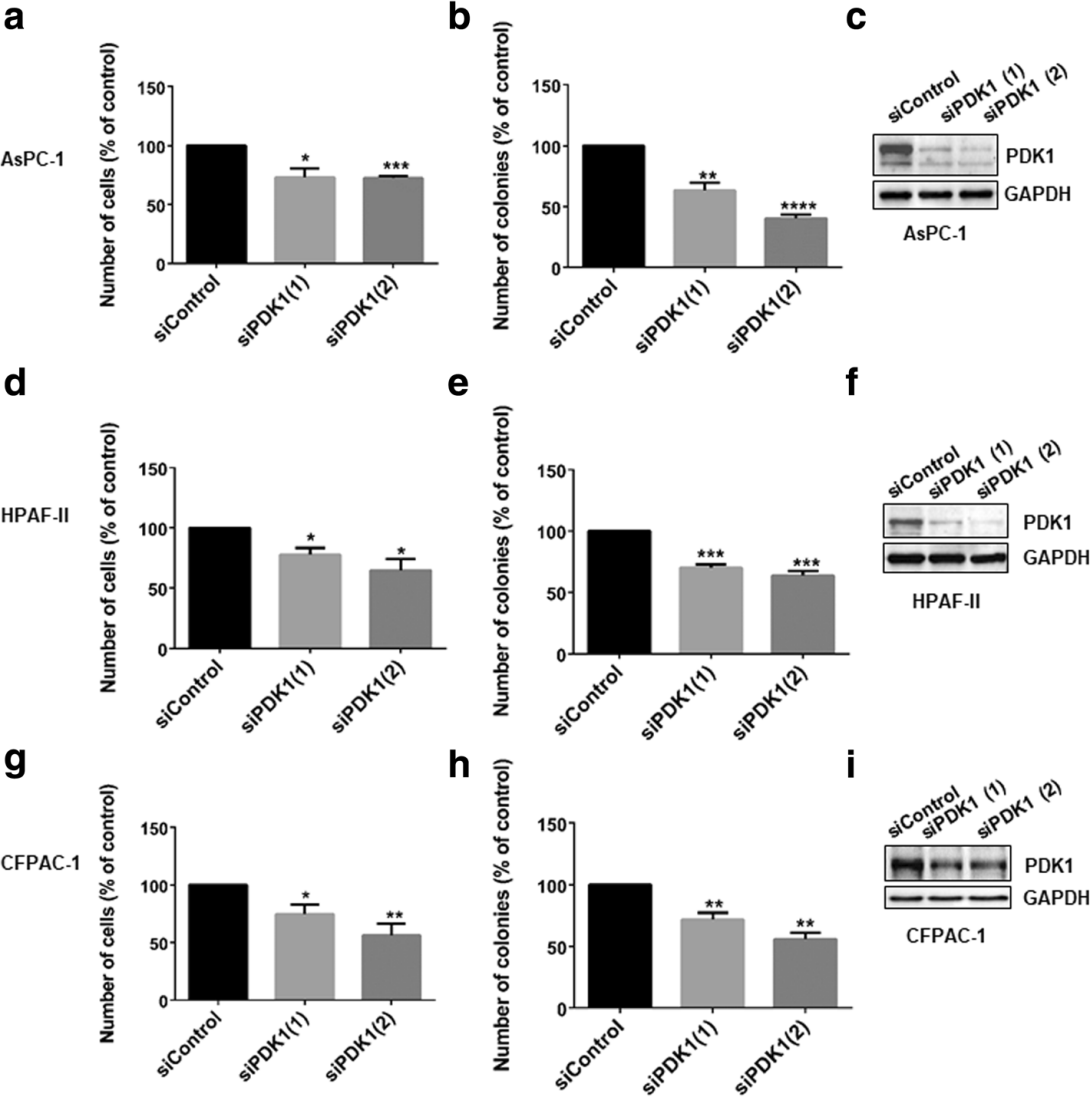
siRNA-mediated PDK1 downregulation inhibits cell proliferation and anchorage-independent growth of PDAC cells. AsPC-1 (**a-c**), HPAF-II (**d-f**) and CFPAC-1 (**g-i**) cells were transfected with two siRNA sequences specifically targeting PDK1 or a non-targeting siRNA (siControl) and then manually counted with trypan blue exclusion (**a,d,g**) or plated on soft agar (**b,e,h**), as specified in the Methods. Data are expressed as percentage of cells transfected with siControl and are means ± SEM of *n* ≥ 3 independent experiments performed in duplicate. T-test and GraphPad Prism version 6.0 were used for statistical analysis, and data from cells transfected with each siRNA were compared to data from cells transfected with siControl. Representative blots confirming efficient PDK1 downregulation are also shown (**c,f,i,**). GAPDH was used as loading control. *p < 0.05, **p < 0.01, ***p < 0.001, ****p < 0.0001

**Fig. 2:**
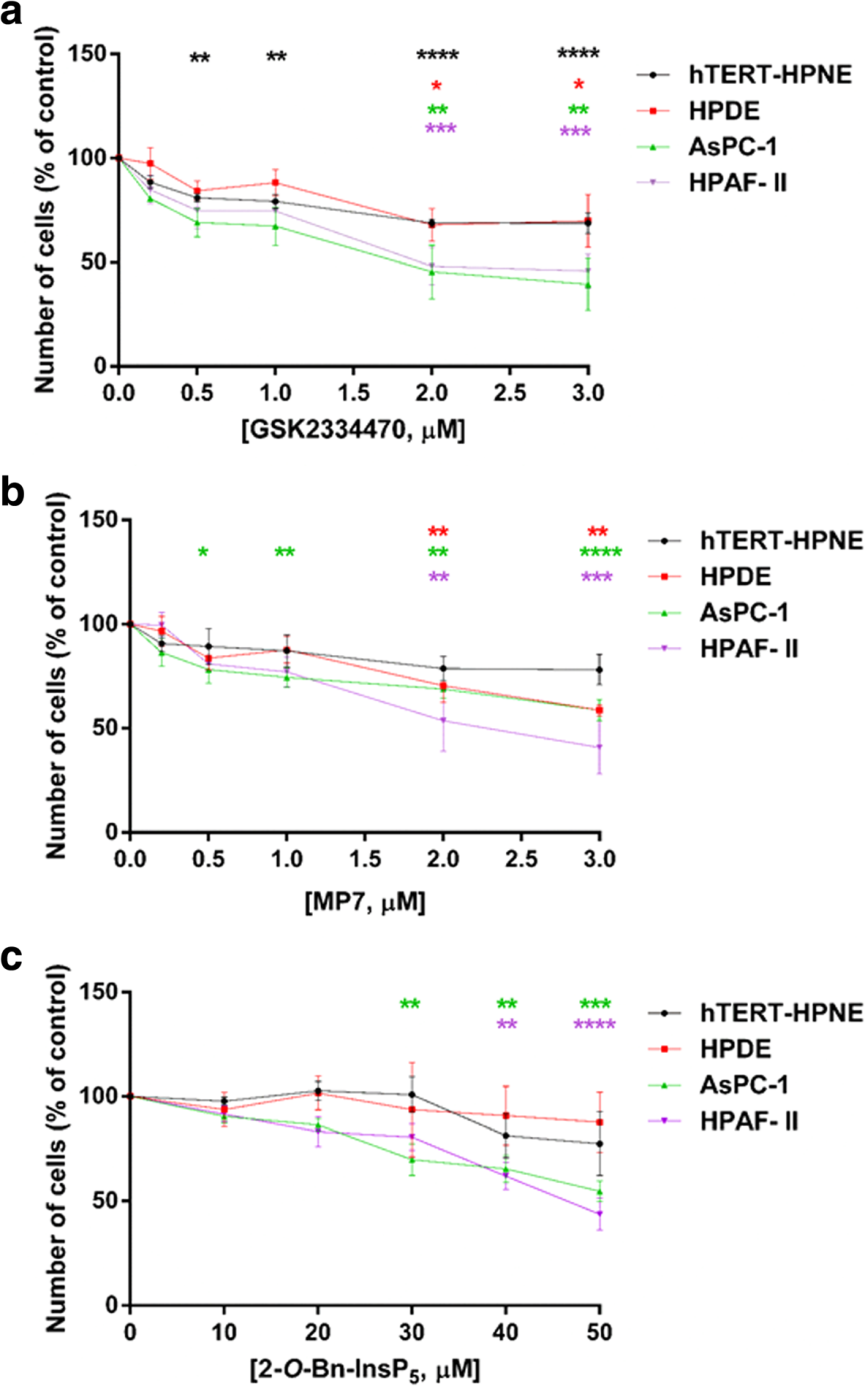
Pharmacological inhibition of PDK1 reduces PDAC cells proliferation in monolayer culture. Non-malignant epithelial pancreatic cells (hTERT-HPNE, HPDE) as well as PDAC cells (AsPC-1, HPAF-II) were treated with increasing concentrations of the PDK1 inhibitors GSK2334470 (**a**), MP7 (**b**) and 2-*O*-Bn-InsP_5_ (**c**) for 72 h, and cell viability was assessed. Data are expressed as percentage of control cells treated with vehicle alone (DMSO) and are means ± SEM of n ≥ 3 independent experiments performed in duplicate. For each cell line, one-way ANOVA with Dunnett’s multiple comparisons test was used for statistical analysis between each treatment and its corresponding DMSO-treated control. Analysis was performed with GraphPad Prism version 6.0. *p < 0.05, **p < 0.01, ***p < 0.001, ****p < 0.0001

**Fig. 3:**
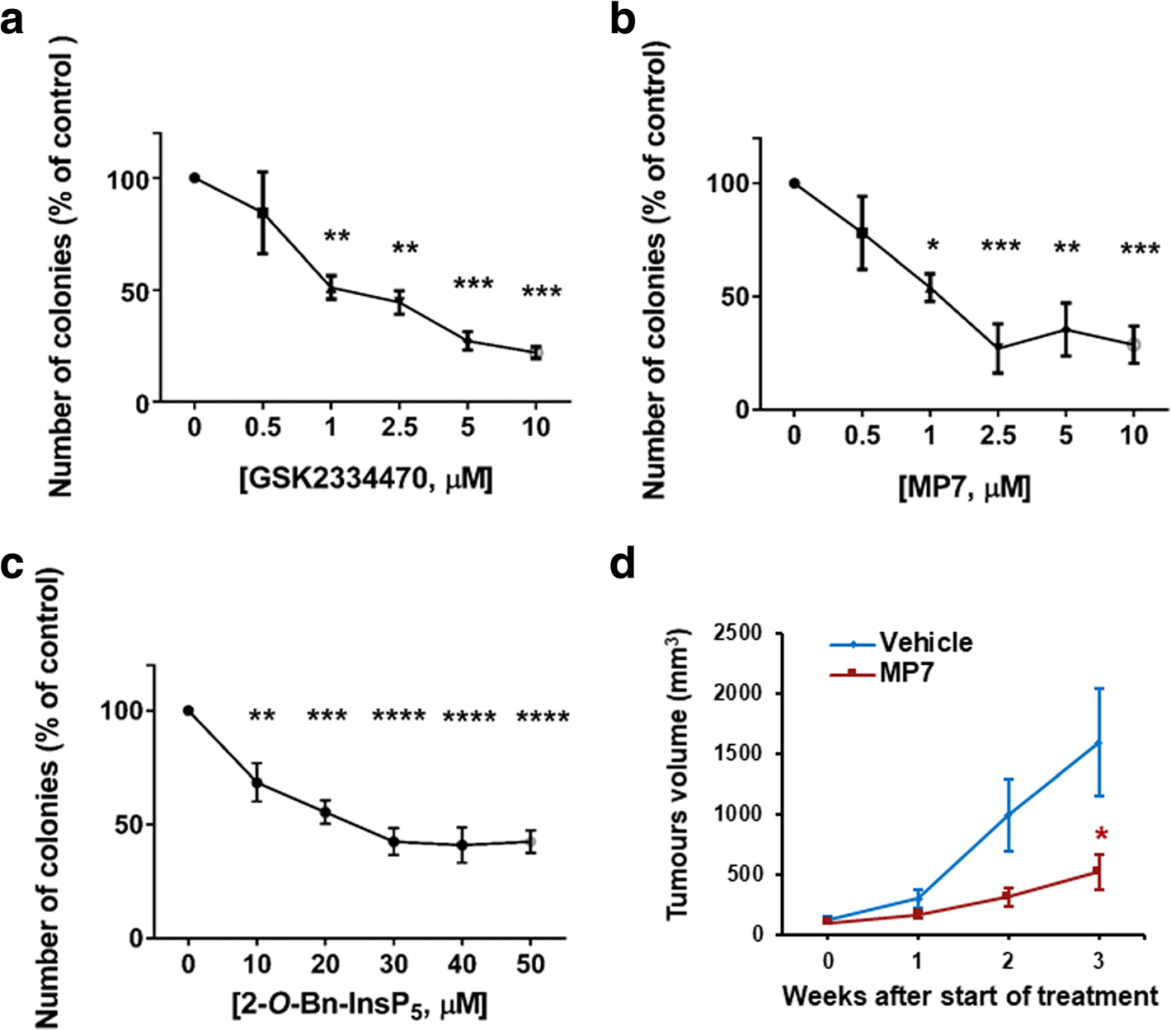
Pharmacological inhibition of PDK1 reduces PDAC cells anchorage independent growth and PDAC cells growth in vivo. **(a-c)** AsPC-1 cells were treated with PDK1 inhibitors and plated on soft agar. After 5 weeks, colonies were stained and counted using ImageJ software. Data are expressed as percentage of control cells treated with DMSO and are means ± SEM of n ≥ 3 independent experiments performed in duplicate. One-way ANOVA with Dunnett’s multiple comparisons test was used for statistical analysis between each treatment and its corresponding DMSO-treated control. Analysis was performed with GraphPad Prism version 6.0. *p < 0.05, **p < 0.01, ***p < 0.001, ****p < 0.0001. **(d)** Mice (*n* = 7/group) were injected with HPAF-II cells and treated as described in the Methods section. Data indicate means ± SEM of tumour volumes at the indicated times after start of treatment with MP7. * *p* < 0.05 as assessed by Student’s t-test

To investigate the specific role of PDK1 in pancreatic cancer proliferation, PDAC cell lines were transiently transfected with two siRNAs specifically targeting the protein, as well as a non-targeting siRNA (“siControl”). Downregulation of PDK1 expression reduced AsPC-1, HPAF-II and CFPAC-1 anchorage-dependent (Fig. 1 a, d, g), and anchorage-independent growth (Fig. 1 b, e, h). Efficient downregulation of PDK1 was confirmed by Western blotting (Fig. 1 c, f, i). We next determined the effect of PDK1 pharmacological inhibition on PDAC cells. Four malignant (HPAF-II, AsPC-1, CFPAC-1, PANC-1) and two non-malignant pancreatic epithelial cell lines (hTERT-HPNE, HPDE) were treated with increasing concentrations of selective PDK1 inhibitors. Specifically, cells were treated with the small molecules GSK2334470 [28] and MP7 [27] as well as the Inositol (1,3,4,5,6) pentakisphosphate (Ins*P*_*5*_) derivative 2-*O-*benzyl*-*InsP_5_ (2-*O-*Bn*-*InsP_5_) that we previously reported to target PDK1 selectively [29]. We observed that treatment of all cell lines with each inhibitor significantly and dose-dependently reduced the number of AsPC-1 and HPAF-II cells compared to control cells treated with vehicle (Fig. 2 a-c). Overall, non-malignant hTERT-HPNE and HPDE cells appeared to be slightly or not sensitive to PDK1 inhibition compared to PDAC cells, with 2-*O*-Bn-InsP_5_ not having any statistically significant effect on hTERT-HPNE and HPDE cell numbers at any tested concentrations (Fig. 2 a-c). Similar results were obtained in CFPAC-1 (Additional file 2: Figure S1a,b) and PANC-1 (Additional file 2: Figure S2a,b). Moreover, treatment of AsPC-1 cells with the three inhibitors significantly reduced their anchorage-independent growth, as assessed by soft agar assays (Fig. 3 a-c, Additional file 2: Figure S3a). PDK1 inhibition also reduced anchorage-independent growth in CFPAC-1 (Additional file 2: Figure S1c,d), PANC-1 (Additional file 2: Figure S2c,d, Additional file 2: Figure S3b-d), and HPAF-II (Additional file 2: Figure S4a,b) cells. It has been reported previously that genetic ablation of *PDPK1* reduces KRas^G12D^- driven PDAC development in a transgenic mouse model and that treatment with a pan class I PI3Ks inhibitor reduces PDAC progression in KPC mice, the animal model that most accurately recapitulates the human disease [26]. Whether pharmacological inhibition of PDK1 could also affect progression of PDAC in vivo has not been previously assessed. To investigate this possibility, HPAF-II cells (3.5 × 10^6^) were injected subcutaneously into the right flank of athymic CD-1 nu/nu mice (5–7 weeks old) and mice were treated with MP7 (75 mg/kg) or vehicle, once xenografts became palpable. No body weight loss was observed during treatments, indicating that MP7 is well tolerated at the dose used for this study. Tumour volumes were monitored every week with the use of a caliper, and volumes were calculated as described in the Methods section. Treatment of mice with MP7 significantly inhibited tumour growth in vivo (Fig. 3d), indicating that targeting PDK1 pharmacologically is able to reduce PDAC progression in vivo.

Taken together these data indicate that PDK1 regulates PDAC cell proliferation and that inhibition of the enzyme can efficiently reduce PDAC growth in vitro. At the same time, our in vivo results, together with previous evidence using genetic approaches, indicate that PDK1 represents a novel important target to counteract PDAC progression, and that MP7 might represent a valuable drug to be used for PDAC treatment.

### PDK1 regulates SGK3 activation in PDAC cells

**Fig. 4:**
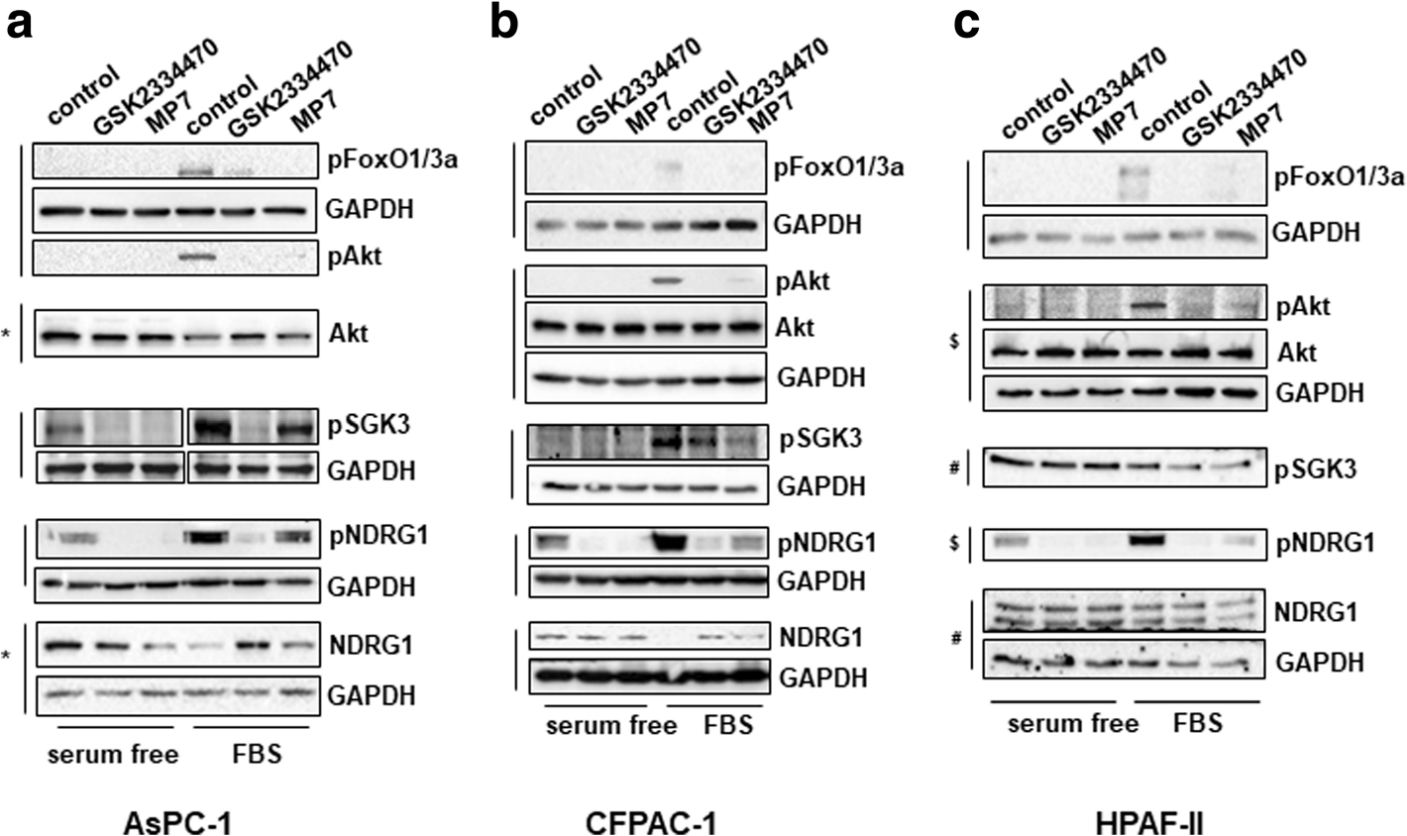
Effect of PDK1 pharmacological inhibition on signalling pathways in PDAC cell lines. AsPC-1 (**a**), CFPAC-1 (**b**) and HPAF-II (**c**) cells were serum starved for 24 h and then treated with GSK2334470 (10μΜ) or MP7 (10μΜ) in the presence or absence of FBS for 1h, prior to cell lysis. Lysates were analysed by Western blotting using the indicated antibodies (details of antibodies are provided in the Methods section). In all blots GAPDH was used as loading control. Representative blots are shown. Vertical lines indicate membranes derived from the same gel. In (**b**,**c**) membranes incubated with anti-pAkt (Thr308) were stripped and re-incubated with anti-Akt. *, ^#^, ^$^ indicate membranes that derived from the same gel (therefore only one GAPDH is shown)

**Fig. 5:**
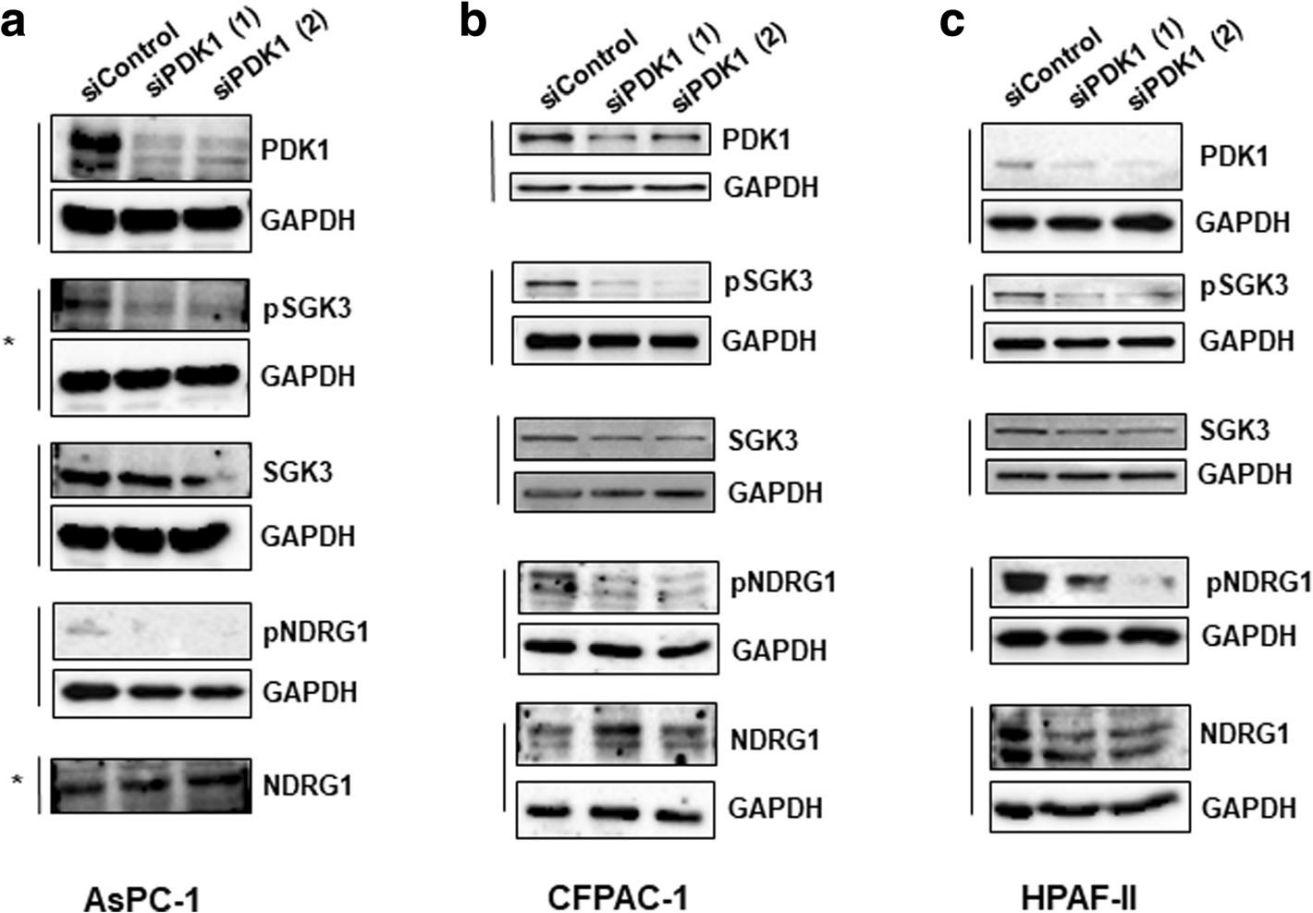
Effect of PDK1 downregulation on signalling pathways in PDAC cell lines. AsPC-1 (**a**), CFPAC-1 (**b**) and HPAF-II (**c**) were transfected with the indicated siRNAs and lysed 72 h post-transfection. Lysates were then analysed by Western blotting using the indicated antibodies. Representative blots are shown. In all blots GAPDH was used as loading control. *indicates membranes that derived from the same gel (therefore only one GAPDH is shown)

In order to gain further insight into the mechanisms involved in the PDK1-dependent regulation of PDAC cell growth, we next analysed the effect of PDK1 inhibition on activation of specific signalling pathways. First, we observed that treatment of AsPC-1 (Fig. 4 a), CFPAC-1 (Fig. 4 b) and HPAF-II (Fig. 4 c) cells with either GSK2334470 or MP7 efficiently blocked the FBS-induced phosphorylation of Akt at its residue Thr308, a bona fide readout of PDK1 activity, without affecting the total levels of Akt. Consistent with reduced Akt activation, inhibition of FBS-mediated FoxO1(Thr24)/FoxO3a(Thr32) phosphorylation was also detected in these cells (Fig. 4 a-c). Since PDK1 has been described to regulate SGK3 activation [30] we next investigated the effect of PDK1 inhibition on SGK3 phosphorylation at its residue Thr320 in PDAC cells. Consistent with data on Akt Thr308, inhibition of PDK1 also reduced SGK3 phosphorylation (Fig. 4 a-c). To investigate further the signalling pathways regulated by PDK1/SGK3 in PDAC cells we next analysed the effect of PDK1 inhibition on the metastasis suppressor N-Myc downstream regulated 1 (NDRG1) which has been recently identified as a downstream target of SGK3 [30]. SGK3 has been reported to regulate NDRG1 phosphorylation at its residue Thr346, which leads to degradation of the protein [30]. A clear inhibition of NDRG1 Thr346 phosphorylation (both in the absence and in the presence of FBS) was detected in AsPC-1 (Fig. 4a), CFPAC-1 (Fig. 4b) and HPAF-II (Fig. 4c) cells upon treatment with both PDK1 inhibitors. Increased protein levels of NDRG1 were observed in FBS-stimulated AsPC-1 (Fig. 4a) and CFPAC-1 (Fig. 4b) cells upon treatment with both PDK1 inhibitors compared to FBS-stimulated, untreated cells. Consistent with data obtained using the two inhibitors, siRNAs-mediated downregulation of PDK1 also clearly reduced SGK3 and NDRG1 phosphorylation in AsPC-1 (Fig. 5a), CFPAC-1 (Fig. 5b) and HPAF-II (Fig. 5c) cells.

**Fig. 6:**
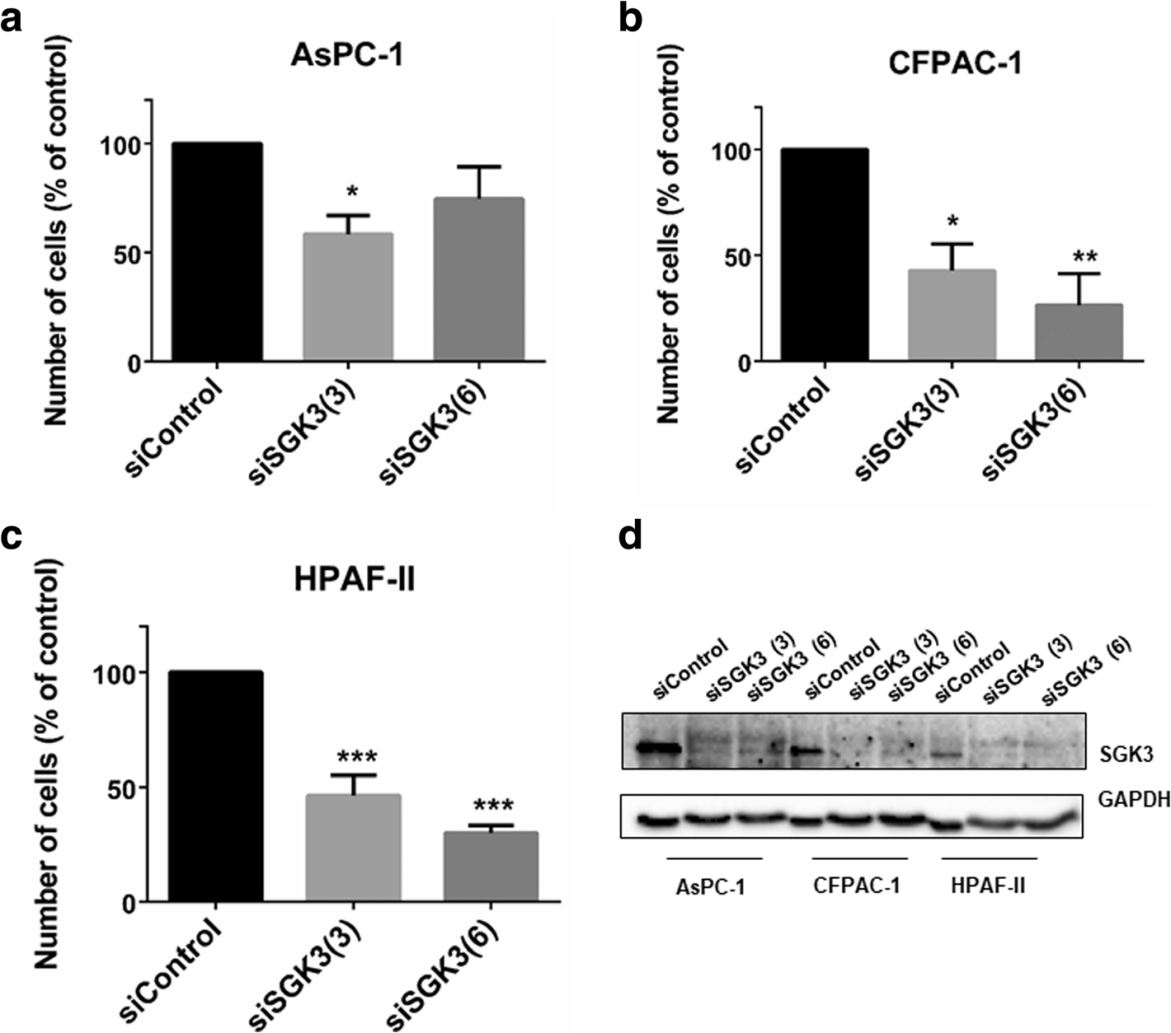
siRNA-mediated SGK3 downregulation inhibits PDAC cells growth**.** AsPC-1 (**a**), CFPAC-1 (**b**) and HPAF-II (**c**) cells were transfected with two siRNAs specifically targeting SGK3 or a non-targeting siRNA (siControl) and then manually counted with trypan blue exclusion as specified in the Methods. Data are expressed as percentage of cells transfected with siControl and are means ± SEM of n ≥ 3 independent experiments performed in duplicate. T-test and GraphPad Prism version 6.0 were used for statistical analysis. *p < 0.05, **p < 0.01, ***p < 0.001. (**d**) Representative blot confirming efficient SGK3 downregulation in all cell lines. GAPDH was used as loading control

Taken together these data indicate that PDK1 regulates both SGK3 and NDRG1 in PDAC cell lines. Interestingly, we noticed that downregulation of SGK3 efficiently reduced the number of AsPC-1 (Fig. 6a), CFPAC-1 (Fig. 6b) and HPAF-II (Fig. 6c) cells, suggesting that inhibition of PDK1 can partly affect PDAC cell proliferation through its effect on SGK3 activation.

### Combination of PDK1 and p110γ inhibitors strongly reduces pancreatic cancer cell growth in vitro

**Fig. 7:**
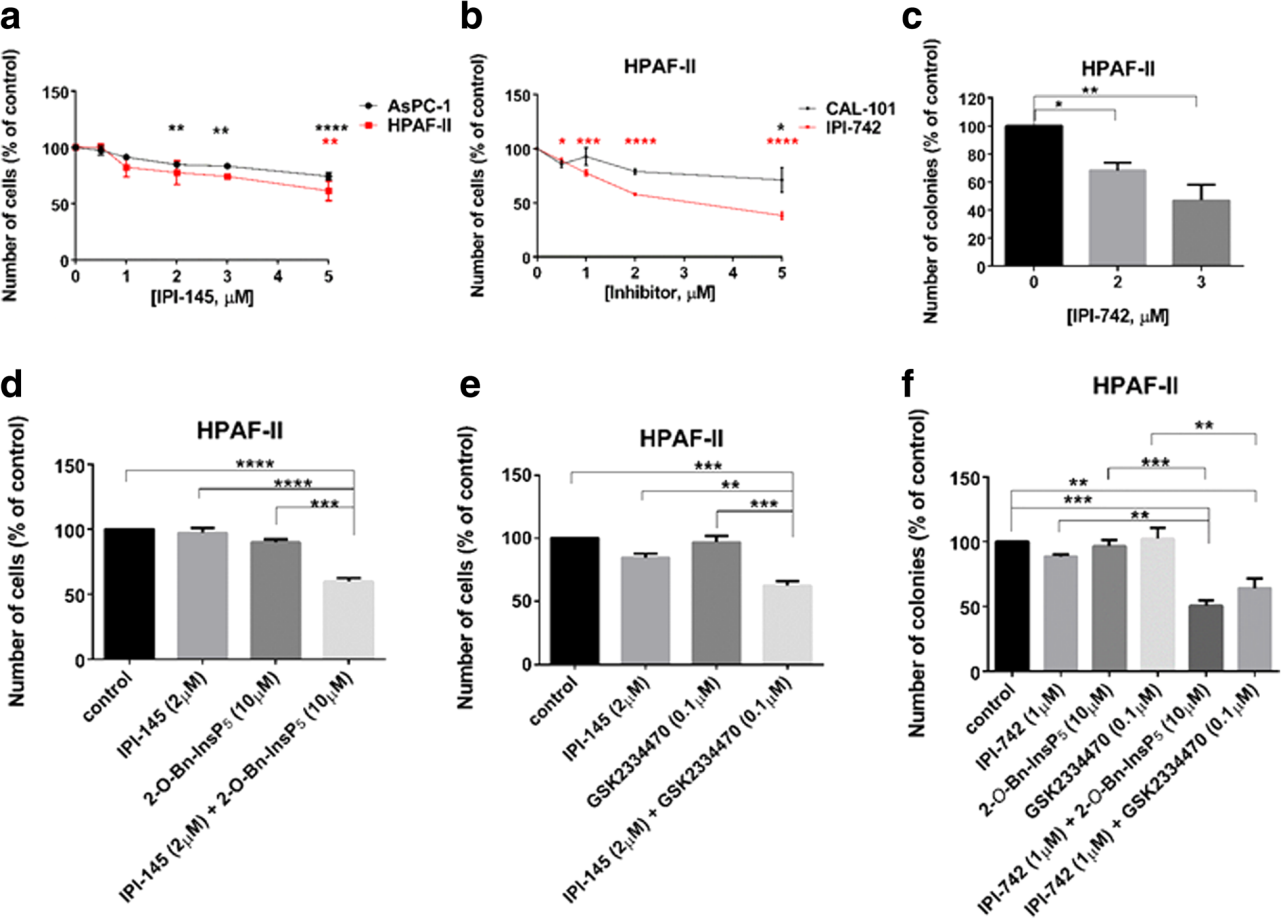
PDK1 inhibitors enhance the effect of p110δ/γ inhibitors on PDAC cell growth**.** (**a,b**) HPAF-II and AsPC-1 cells were treated with the indicated concentrations of the p110δ/γ inhibitor IPI-145 (**a**). Alternatively, HPAF-II cells were treated with the indicated concentrations of the p110δ/γ inhibitor IPI-742 or the selective p110δ inhibitor CAL-101 (**b**). The number of cells was assessed after 72 h. Data are expressed as percentage of cells treated with vehicle (control) and are means ± SEM of *n* = 3 independent experiments performed in duplicate. *p < 0.05, **p < 0.01, ***p < 0.001, ****p < 0.0001 vs control cells. (**c**) HPAF-II cells were plated on soft agar and treated with the indicated concentrations of IPI-742. Data are expressed as percentage of colonies from cells treated with vehicle (control) and are means ± SEM of *n* = 4 independent experiments performed in duplicate. *p < 0.05, **p < 0.01 vs control. (**d, e**) HPAF-II cells were treated with IPI-145 (2 μM), 2-*O*-Bn-InsP_5_ (10 μM), GSK2334470 (0.1 μM) or the indicated combinations. The number of cells was assessed after 72 h. Data are expressed as percentage of cells treated with vehicle (control) and are means ± SEM of n = 3 independent experiments performed in duplicate. **p < 0.01, ***p < 0.001, ****p < 0.0001. (**f**) HPAF-II cells were plated on soft agar and treated with IPI-742 (1 μM), 2-*O*-Bn-InsP_5_ (10 μM), GSK2334470 (0.1 μM) or the indicated combinations. Data are expressed as percentage of colonies from cells treated with vehicle (control) and are means ± SEM of *n* = 3 independent experiments performed in duplicate. **p < 0.01, ***p < 0.001

Previous work in our laboratory demonstrated a key role for the PI3K isoform p110γ in PDAC cell proliferation [5]. Consistent with our previous report, we observed that treatment of HPAF-II and AsPC-1 cells with the p110δ/γ inhibitor IPI-145 (Infinity Pharmaceuticals, Inc.) significantly and dose dependently reduced cell growth (Fig. 7a). Similarly, treatment with the more potent p110δ/γ inhibitor IPI-742 (Infinity Pharmaceuticals, Inc.) reduced cell growth (Fig. 7b) and colony formation (Fig. 7c) in HPAF-II cells. Reduced cell growth was also detected in AsPC-1 cells upon treatment with IPI-742 (Additional file 2: Figure S5a). Importantly, the detected inhibition was mainly due to the effect of the compound on p110γ as in the same experimental conditions the specific p110δ inhibitor CAL-101 affected cell numbers only slightly when used at the highest concentration in HPAF-II (Fig. 7b) while it did not affect AsPC-1 cells at any of the concentrations used (Additional file 2: Figure S5a). Consistent with a specific role for p110γ, treatment with IPI-549 (Selleckchem), a novel and specific p110γ inhibitor, reduced anchorage-dependent growth of PDAC cells without affecting the non-malignant pancreatic epithelial cell lines (Additional file 2: Figure S5b). Reduced anchorage-independent growth was also detected in AsPC-1 (Additional file 2: Figure S5c, d) and PANC-1 (Additional file 2: Figure S5c) cells upon treatment with IPI-549. Interestingly, we observed that combination of IPI-145 with 2-*O*-Bn-InsP_5_ (Fig. 7d) or GSK2334470 (Fig. 7e) used at sub-optimal concentrations resulted in enhanced reduction of HPAF-II cell numbers. Consistent with this, combination of GSK2334470 with IPI-742 (Additional file 2: Figure S6a) reduced numbers of AsPC-1 cells more potently than each inhibitor used at sub-optimal concentrations. A similar trend was observed in AsPC-1 cells upon combination of IPI-549 with GSK2334470 (Additional file 2: Figure S6b) and in CFPAC-1 cells upon combination of IPI-549 with either GSK2334470 or 2-*O*-Bn-InsP_5_ (Additional file 2: Figure S6c). Finally, we analysed the effect of these combinations on anchorage-independent growth. Data indicated that treatment with IPI-742 in combination with either GSK2334470 or 2-*O*-Bn-InsP_5_ strongly reduced the number of HPAF-II colonies in soft agar assays, when sub-optimal concentrations of each inhibitor were used (Fig. 7f). A similar trend was observed in AsPC-1 (Additional file 2: Figure S6d) and CFPAC-1 (Additional file 2: Figure S6e) cells when 2-*O*-Bn-InsP_5_ was combined with IPI-549.

Taken together, these data indicate that simultaneous inhibition of the PI3K isoform p110γ and PDK1 affects PDAC cell growth more potently than inhibition of p110γ or PDK1 alone, indicating that combination of drugs targeting the two proteins can enhance their effect.

## Discussion

In this study, we identified PDK1 as a novel potential therapeutic target in PDAC. First, our data demonstrated that downregulation of the protein using either pharmacological inhibitors or specific siRNAs reduced PDAC cell numbers and colonies formation in soft agar assays, indicating that PDK1 plays a central role in regulation of PDAC cell growth. Our results are consistent with a previous study reporting that pancreas-specific deletion of *PDPK1* reduced acinar-to-ductal metaplasia, pancreatic intraepithelial neoplasia formation and PDAC formation in a KRas^G12D^-driven transgenic model [26]. Interestingly, ablation of *PDPK1*1 did not affect lung tumour formation in KRas^G12D^-driven models of non small cell lung carcinoma [26], suggesting a specific role for the enzyme during PDAC development. Whether PDK1 might act specifically downstream of mutant KRas in the context of pancreatic cancer remains to be established [31]. In this respect, it is worth mentioning that all PDAC cell lines used in our study bear a KRas mutation in G12 [32], possibly providing further evidence of a specific KRas/PDK1 pathway in PDAC [31].

In an effort to define the mechanisms of PDK1-mediated regulation of PDAC cell growth, we observed that PDK1 regulated both SGK3 and its downstream effector NDRG1 in PDAC cell lines. Furthermore, downregulation of SGK3 using selective siRNAs reduced PDAC cell numbers, strongly suggesting that PDK1 regulates PDAC cell growth through SGK3 activation, at least partly. These data are consistent with accumulating evidence indicating that PDK1 can contribute to cancer through activation of several downstream effectors. Indeed, while for many years the potential role of this enzyme in cancer was almost exclusively associated with its regulatory role on Akt activation, several recent data have revealed additional roles for PDK1, independently of Akt activation [24, 25]. For instance, it was reported that constitutively active Akt was not able to rescue the reduced anchorage-independent growth or the increased apoptosis resulting from downregulation of PDK1 in breast cancer cells MDA-MB-231 [13]. Importantly, SGK3, that can be regulated through PDK1-dependent phosphorylation of residue Thr320 within its T-loop, has been identified as a key regulator of such PI3K/PDK1-dependent, Akt-independent signalling pathways in cancer [33]. Evidence includes a study demonstrating that PDK1-mediated SGK3 activation was critical for anchorage-independent growth in a subset of *PIK3CA* (the gene encoding for p110α) mutant breast cancer cell lines with minimal Akt activation [23]. Similarly, SGK3 was implicated in Akt-independent oncogenic signalling [33]. Our data strongly suggest that SGK3 is involved in regulation of PDAC cell growth downstream of PDK1. Whether PDK1 controls PDAC growth solely through SGK3 activation, in an Akt-independent mechanism, remains to be established.

Our study further demonstrates that pharmacological inhibition of PDK1, using three distinct chemical compounds, strongly reduced both anchorage-dependent and anchorage-independent PDAC cell growth in vitro*.* Importantly, we also show that chemical inhibition of PDK1 reduced growth of PDAC cells in vivo.

Several lines of evidence now support the conclusion that PDK1 is an important potential therapeutic target in different cancer types and that its inhibition can prove beneficial to reduce growth of several cancer cell types. For instance, we reported that inhibition of PDK1 with 2-*O*-Bn-InsP_5_ was able to reduce cell numbers in different cancer cell lines as well as growth of prostate cancer PC3 cells in a xenograft model in nude mice [29]. Similarly, MP7 reduced soft agar colony formation in a subset of cancer cell lines as well as primary tumour xenograft lines [34]. Results from our study further supports the conclusion that PDK1 represents an important molecular target to develop novel therapeutic anti-cancer strategies. More importantly, our results indicate that inhibition of PDK1 can represent a useful strategy to counteract progression of PDAC. As there are very few treatments available for pancreatic cancer patients which provide a very limited increase in survival, these results might represent an important step towards the identification of novel, much needed, therapeutic options for this deadly disease.

Finally, we report that inhibition of PDK1 potentiates the effect of other drugs in PDAC cell lines. We previously demonstrated that the class IB PI3K isoform p110γ is overexpressed in PDAC and it has a critical role in PDAC cell proliferation [5]. Here we show that combination of selective p110γ inhibitors with PDK1-targeting compounds reduced anchorage-dependent and independent-growth of PDAC cells more potently than each treatment alone, when used at sub-optimal concentrations. It remains to be established whether p110γ and PDK1 act on the same or on distinct signalling cascades to regulate PDAC growth. Indeed, the enhanced effect of the combined treatment might be the result of full inhibition of the same signalling pathway, as opposed to the effect of each compound alone that, used at sub-optimal concentrations, would inhibit the pathway only partially. On the other hand, we cannot rule out the possibility that the combination of the two classes of inhibitors can overcome mechanisms of resistance and result in more pronounced inhibition of PDAC cell growth. In this respect, it is worth mentioning that several lines of evidence now suggest that PDK1 can have a role in cancer chemoresistance and that its inhibition can promote chemosensitization [35]. Interestingly, SGK3 has been linked to development of mechanism of resistance to PI3K and Akt inhibitors [36] therefore it is tempting to speculate that the additive effect that we detected by using combination of p110γ and PDK1 inhibitors might be due to inhibition of potential PDK1/SGK3-mediated intrinsic mechanisms of resistance to p110γ inhibitors. Additional studies are now required to ascertain whether combination of p110γ and PDK1 inhibitors would prove to be more efficient than each single agent administration in in vivo models of PDAC.

## Conclusions

In summary, in this study we demonstrated that inhibition of PDK1 reduces PDAC cell growth in vitro and in vivo. These results, together with previous evidence using genetic ablation of *PDPK1*, provide a strong rationale to investigate further the use of PDK1 inhibitors in PDAC as novel therapeutic strategies for pancreatic cancer patients. Our data further suggest that combination of PDK1 inhibitors with selective PI3K inhibitors might enhance their anti-cancer activity, possibly by targeting SGK3-dependent resistance mechanisms.

## Additional file

Additional file 1: Synthetic procedure followed for the synthesis of MP7 and characterization of intermediates. (DOCX 129 kb)

Additional file 2: **Figure S1.** Effect of pharmacological inhibition of PDK1 on CFPAC-1 cells. CFPAC-1 cells were treated with different concentrations of PDK1 inhibitors and their effects on cell viability (a, b) and anchorage independent growth (c, d) were assessed. Data are expressed as percentage of control cells treated with DMSO and are means ± SEM of *n* ≥ 3 independent experiments performed in duplicate. Statistical analysis was performed using GraphPad Prism version 6.0 and one-way ANOVA with Dunnett’s multiple comparisons test. *p < 0.05, **p < 0.01, ***p < 0.001, ****p < 0.0001 vs control. **Figure S2.** Effect of pharmacological PDK1 inhibition on PANC-1 cells. PANC-1 cells were treated with different concentrations of PDK1 inhibitors and their effects on cell viability (a, b) and anchorage independent growth (c, d) were assessed. Data are expressed as percentage of control cells treated with DMSO and are means ± SEM of *n* ≥ 3 independent experiments performed in duplicate. Statistical analysis was performed using GraphPad Prism version 6.0 and one-way ANOVA with Dunnett’s multiple comparisons test. *p < 0.05, **p < 0.01, ***p < 0.001, ****p < 0.0001 vs control. **Figure S3.** Representative images of 3D colonies of AsPC-1 and PANC-1 cells treated with PDK1 inhibitors. Images of AsPC-1 colonies treated with different concentrations of GSK2344470 (a) as well as PANC-1 colonies treated with MP7 (b) and GSK2344470 (c) were acquired using 4X magnification lens. (d) Images of the 6-well plates of PANC-1 colonies treated with GSK2344470 (left) and MP7 (right), as visualized by the ChemiDoc system (BioRad). **Figure S4.** Effect of pharmacologicalinhibition of PDK1 on HPAF-II cells anchorage–independent growth. HPAF-II cells were treated with the indicated concentrations of the PDK1 inhibitors GSK2334470, 2-*O*-Bn-InsP_5_ (a) and MP7 (b) and their effects on anchorage-independent growth were determined. Data are expressed as percentage of control cells treated with DMSO. Data in (a) are means ± SEM of *n* = 3 independent experiments performed in duplicate. Statistical analysis was performed using GraphPad Prism version 6.0 and one-way ANOVA with Dunnett’s multiple comparisons test. *p < 0.05, **p < 0.01. Data in (b) are means of *n* = 2 independent experiments performed in duplicate. **Figure S5.** Effect of p110δ/γ and p110γ inhibition on PDAC cells. (a,b) AsPC-1 cells were treated with the indicated concentrations of the p110δ/γ inhibitor IPI-742 or the selective p110δ inhibitor CAL-101 (a). Alternatively, PDAC cells AsPC-1 and HPAF-II, together with the two non-malignant epithelial pancreatic cell lines hTERT-HPNE and HPDE, were treated with increasing concentrations of the selective p110γ inhibitor IPI-549 (b). Cell viability was assessed after 72 h. Data are expressed as percentage of cells treated with vehicle alone and are means ± SEM of *n* ≥ 3 independent experiments performed in duplicate. Statistical analysis was performed using GraphPad Prism version 6.0 and one-way ANOVA with Dunnett’s multiple comparisons test. *p < 0.05, **p < 0.01, ****p < 0.0001 vs control. (c) AsPC-1 and PANC-1 were plated on soft agar and treated with the indicated concentrations of IPI-549. Data are expressed as percentage of cells treated with vehicle alone and are means ± SEM of n ≥ 3 independent experiments performed in duplicate. Statistical analysis was performed using GraphPad Prism version 6.0 and one-way ANOVA with Dunnett’s multiple comparisons test. *p < 0.05, **p < 0.01, ***p < 0.001, ****p < 0.0001 vs corresponding control. (d) Representative images of the effect of different concentrations of IPI-549 on AsPC-1 cells colony formation (4X magnification lens). **Figure S6.** PDK1 inhibitors enhance the effect of p110δ/γ inhibitors. (a-c) AsPC-1 (a,b) and CFPAC-1 (c) cells were treated with the indicated inhibitors alone or in combination. Cell viability was assessed after 72 h. Data are expressed as percentage of cells treated with vehicle alone and are means ± SEM of n ≥ 3 independent experiments performed in duplicate. Statistical analysis was performed using GraphPad Prism version 6.0 and one-way ANOVA with Dunnett’s multiple comparisons test. *p < 0.05, **p < 0.01, ***p < 0.001. (d,e) AsPC-1 (d) and CFPAC-1 (e) cells were plated on soft agar and treated with the indicated inhibitors and their combination. Data are from *n* = 2 independent experiments performed in duplicate. (ZIP 2460 kb)

## Abbreviations

2-*O-*Bn*-*InsP_5_: 2-*O-*benzyl*-*Inositol (1,3,4,5,6) pentakisphosphate
EGF: Epidermal growth factor
HCC: human hepatocellular carcinoma
NDRG1: N-Myc downstream regulated 1
PDAC: pancreatic ductal adenocarcinoma
PDK1: 3-phosphoinositide-dependent protein kinase 1
PI3K: phosphoinositide 3-kinase
PKB / Akt: protein kinase B
PtdIns(3,4,5)*P*_*3*_: phosphatidylinositol 3,4,5-trisphosphate: phosphatidylinositol 3,4,5-trisphosphate
PtdIns (4, 5) *P*_*2*_: phosphatidylinositol 4,5-bisphosphate
PTEN: phosphatase and tensin homolog
SGK: serum and glucocorticoid-induced protein kinase.
