# Supplementary material for: Preclinical validation of 3-phosphoinositide-dependent protein kinase 1 inhibition in pancreatic cancer
Source: J Exp Clin Cancer Res. 2019 May 14;38:191. doi: 10.1186/s13046-019-1191-2 (PMC6518649; doi:10.1186/s13046-019-1191-2)
Supplement: Supplementary file 1 — Synthetic procedure followed for the synthesis of MP7 and characterization of intermediates. (DOCX 129 kb) [file 13046_2019_1191_MOESM1_ESM.docx]

The PDK1 inhibitor MP7 was synthetized as reported by Erlanson et al [1]. Only characterization data not yet published are reported here.

**Scheme 1^a^:**

**Reagents and conditions ^a^: *I.*** TBTU, DIPEA, dry DMF, rt, 16 h; ***II.*** 5-fluoro-2-nitroaniline, NaH, DMF, rt, 16 h; ***III.*** Zn, HCl, MeOH–*i*PrOH (1:2), reflux, 2 h; ***IV.*** CDI, dry DMF, 60 °C, 4 h.

**Compound 4:**

**^1^H NMR (CDCl_3_)**: δ 4.30-4.40 (m, 2H, CH_2_OH); 5.16 (d, 1H, *J* = 14.8 Hz, CH); 5.20 (d, 1H, *J* = 14.8 Hz, CH); 5.56-5.61 (m, 1H, CH), 6.26 (d, 1H, *J* = 2.4 Hz, Ar); 6.32 (dd, 1H, *J* = 2.4, 9.6 Hz, Ar); 6.46 (t, 1H, *J* = 6.9 Hz, Ar); 7.04-7.05 (m, 1H, Ar); 7.14-7.21 (m, 2H, Ar); 7.30-7.34 (m, 2H, Ar); 7.38-7.41 (m, 2H, Ar); 7.45-7.48 (m, 2H, Ar); 7.53 (dd, 1H, *J* = 2.3, 6.9 Hz, Ar); 8.05 (d, 1H, *J* = 9.6 Hz; Ar); 8.56 (dd, 1H, *J* = 2.2, 6.9 Hz, Ar), 10.51 (d, 1H, *J* = 7.6 Hz, NH) ppm.

**Compound 5:**

**^1^H NMR (CDCl_3_)**: δ 4.16-4.24 (m, 2H, CH_2_); 5.10-5.22 (m, 2H, CH_2_); 5.50-5.55 (m, 1H, CH); 6.27 (dd, 1H, *J* = 2.4, 8.4 Hz, Ar); 6.35 (d, 1H, *J* = 2.4 Hz, Ar); 6.41 (t, 1H, *J* = 6.8 Hz, Ar); 6.59 (d, 1H, *J* = 8.4 Hz, Ar); 7.02-7.05 (m, 1H, Ar); 7.12-7.19 (m, 2H, Ar); 7.24-7.28 (m, 1H, Ar); 7.24-7.28 (m, 2H, Ar); 7.45-7.49 (m, 3H, Ar); 8.52 (dd, 1H, *J* = 2.0, 7.2 Hz, Ar); 10.47 (d, 1H, *J* = 7.6 Hz, NH) ppm.

**MP7:**

**^13^C NMR (CDCl_3_)**: δ 163.54; 162.15; 156.86; 154.40; 151.76; 149.34; 144.34; 140.65; 139.48; 132.49; 129.90; 128.80; 127.80; 127.29; 124.33; 123.50; 121.97; 118.05; 117.40; 109.72; 108.74; 107.36; 97.74; 71.76; 53.24; 52.03 ppm.

**^19^F NMR (CDCl_3_)**: δ - 136.01 (d, 1F, J = 24 Hz); - 137.60 (d, 1F, J = 24 Hz) ppm.

MP7 was also subjected to FRET-based Z′-Lyte assay (Invitrogen) against PDK1 direct, showing an IC_50_ value of 10.8 nM.

1. Erlanson, D.A., et al., *Discovery of a potent and highly selective PDK1 inhibitor via fragment-based drug discovery.* Bioorg Med Chem Lett, 2011. **21**(10): p. 3078-83.
