# Supplementary figures and images for: Preclinical validation of 3-phosphoinositide-dependent protein kinase 1 inhibition in pancreatic cancer
Source: J Exp Clin Cancer Res. 2019 May 14;38:191. doi: 10.1186/s13046-019-1191-2 (PMC6518649; doi:10.1186/s13046-019-1191-2)

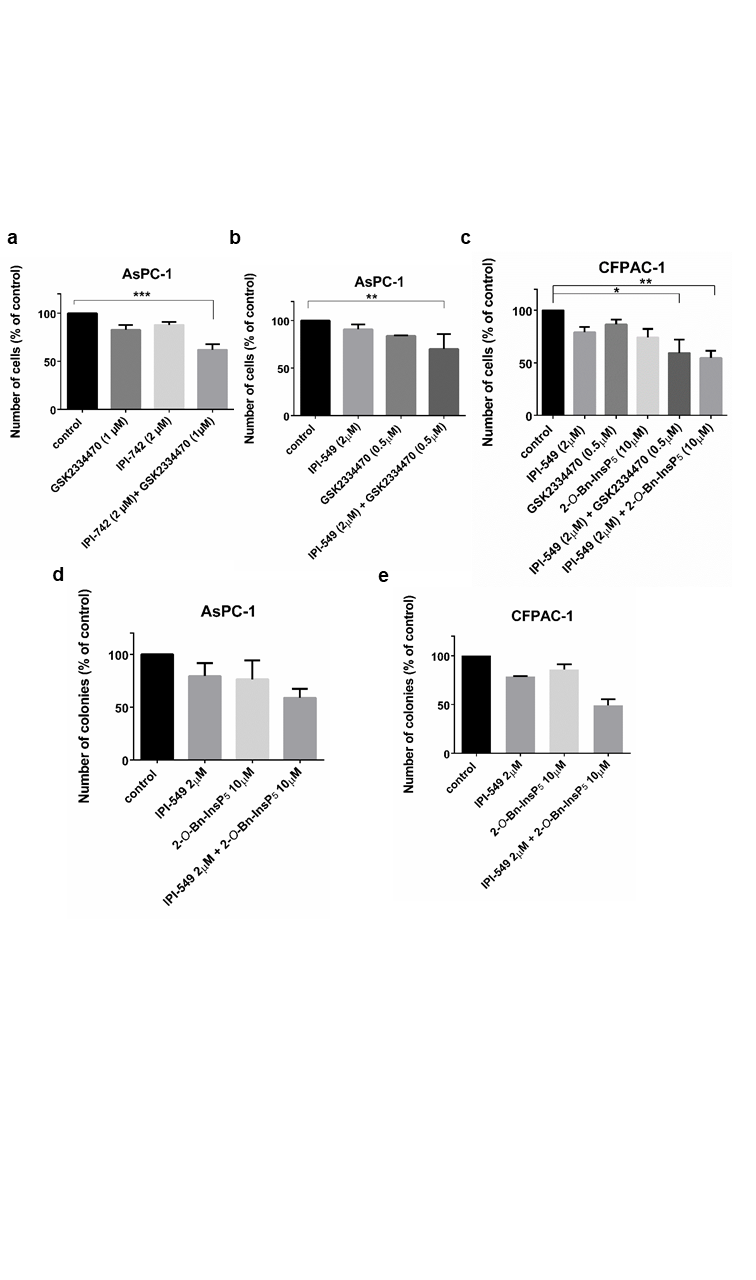

Supplement: Supplementary file 2 — Figure S1. Effect of pharmacological inhibition of PDK1 on CFPAC-1 cells. CFPAC-1 cells were treated with different concentrations of PDK1 inhibitors and their effects on cell viability (a, b) and anchorage independent growth (c, d) were assessed. Data are expressed as percentage of control cells treated with DMSO and are means ± SEM of n ≥ 3 independent experiments performed in duplicate. Statistical analysis was performed using GraphPad Prism version 6.0 and one-way ANOVA with Dunnett’s multiple comparisons test. *p < 0.05, **p < 0.01, ***p < 0.001, ****p < 0.0001 vs control. Figure S2. Effect of pharmacological PDK1 inhibition on PANC-1 cells. PANC-1 cells were treated with different concentrations of PDK1 inhibitors and their effects on cell viability (a, b) and anchorage independent growth (c, d) were assessed. Data are expressed as percentage of control cells treated with DMSO and are means ± SEM of n ≥ 3 independent experiments performed in duplicate. Statistical analysis was performed using GraphPad Prism version 6.0 and one-way ANOVA with Dunnett’s multiple comparisons test. *p < 0.05, **p < 0.01, ***p < 0.001, ****p < 0.0001 vs control. Figure S3. Representative images of 3D colonies of AsPC-1 and PANC-1 cells treated with PDK1 inhibitors. Images of AsPC-1 colonies treated with different concentrations of GSK2344470 (a) as well as PANC-1 colonies treated with MP7 (b) and GSK2344470 (c) were acquired using 4X magnification lens. (d) Images of the 6-well plates of PANC-1 colonies treated with GSK2344470 (left) and MP7 (right), as visualized by the ChemiDoc system (BioRad). Figure S4. Effect of pharmacologicalinhibition of PDK1 on HPAF-II cells anchorage–independent growth. HPAF-II cells were treated with the indicated concentrations of the PDK1 inhibitors GSK2334470, 2-O-Bn-InsP5 (a) and MP7 (b) and their effects on anchorage-independent growth were determined. Data are expressed as percentage of control cells treated with DMSO. Data in (a) are means ± SEM o [file 13046_2019_1191_MOESM2_ESM.zip › Emmanouilidi et al Addtional file 2 S6.tif]

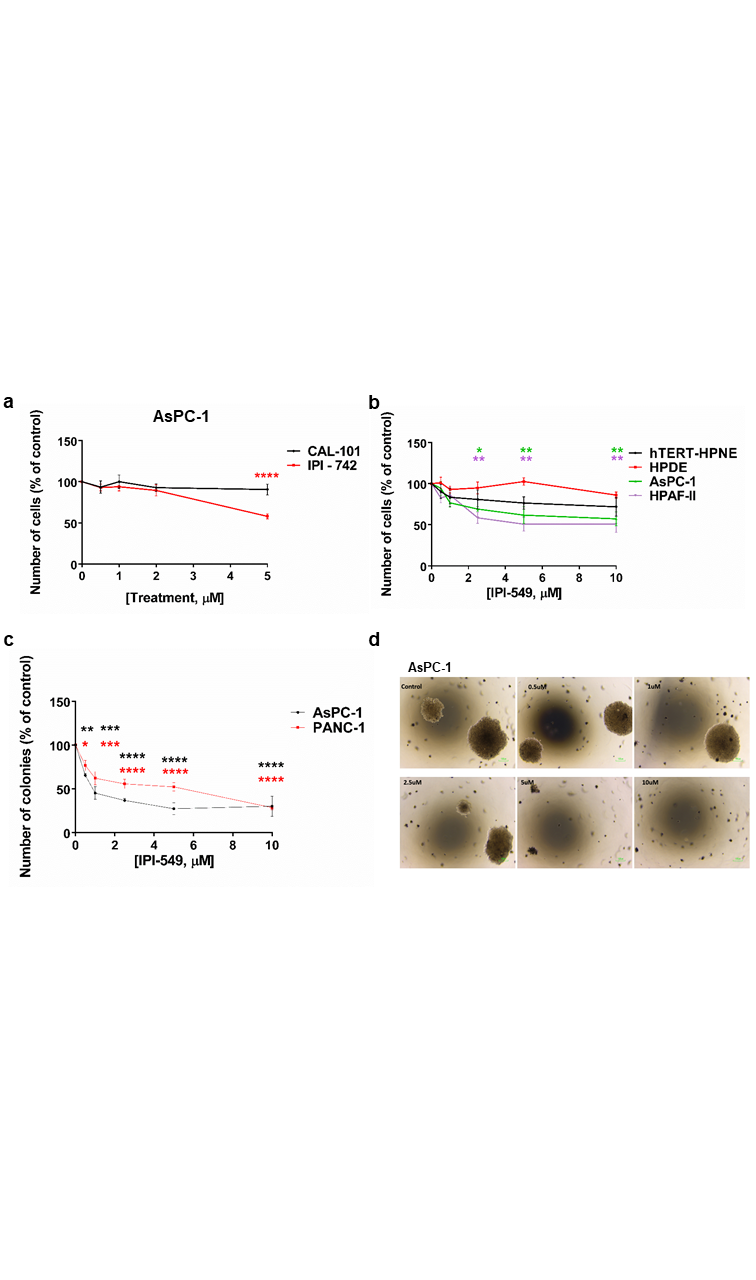

Supplement: Supplementary file 2 — Figure S1. Effect of pharmacological inhibition of PDK1 on CFPAC-1 cells. CFPAC-1 cells were treated with different concentrations of PDK1 inhibitors and their effects on cell viability (a, b) and anchorage independent growth (c, d) were assessed. Data are expressed as percentage of control cells treated with DMSO and are means ± SEM of n ≥ 3 independent experiments performed in duplicate. Statistical analysis was performed using GraphPad Prism version 6.0 and one-way ANOVA with Dunnett’s multiple comparisons test. *p < 0.05, **p < 0.01, ***p < 0.001, ****p < 0.0001 vs control. Figure S2. Effect of pharmacological PDK1 inhibition on PANC-1 cells. PANC-1 cells were treated with different concentrations of PDK1 inhibitors and their effects on cell viability (a, b) and anchorage independent growth (c, d) were assessed. Data are expressed as percentage of control cells treated with DMSO and are means ± SEM of n ≥ 3 independent experiments performed in duplicate. Statistical analysis was performed using GraphPad Prism version 6.0 and one-way ANOVA with Dunnett’s multiple comparisons test. *p < 0.05, **p < 0.01, ***p < 0.001, ****p < 0.0001 vs control. Figure S3. Representative images of 3D colonies of AsPC-1 and PANC-1 cells treated with PDK1 inhibitors. Images of AsPC-1 colonies treated with different concentrations of GSK2344470 (a) as well as PANC-1 colonies treated with MP7 (b) and GSK2344470 (c) were acquired using 4X magnification lens. (d) Images of the 6-well plates of PANC-1 colonies treated with GSK2344470 (left) and MP7 (right), as visualized by the ChemiDoc system (BioRad). Figure S4. Effect of pharmacologicalinhibition of PDK1 on HPAF-II cells anchorage–independent growth. HPAF-II cells were treated with the indicated concentrations of the PDK1 inhibitors GSK2334470, 2-O-Bn-InsP5 (a) and MP7 (b) and their effects on anchorage-independent growth were determined. Data are expressed as percentage of control cells treated with DMSO. Data in (a) are means ± SEM o [file 13046_2019_1191_MOESM2_ESM.zip › Emmanouilidi et al Addtional file 2 S5.tif]

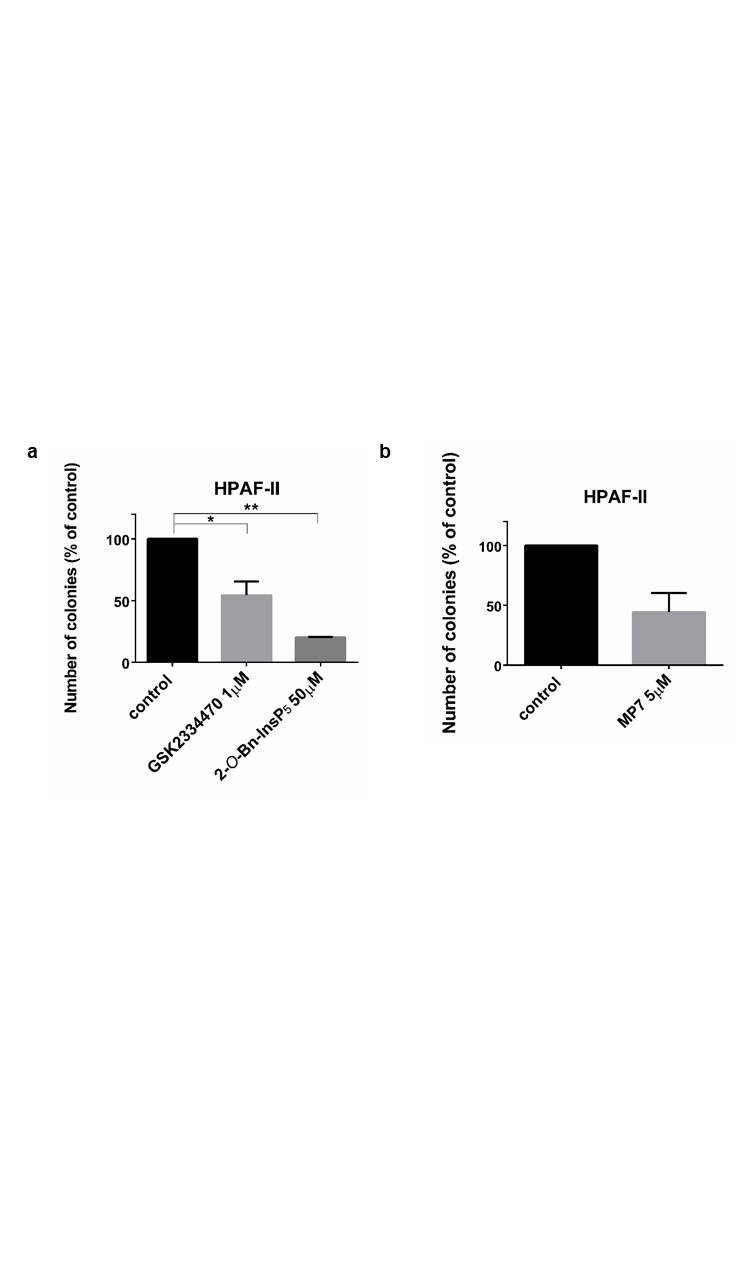

Supplement: Supplementary file 2 — Figure S1. Effect of pharmacological inhibition of PDK1 on CFPAC-1 cells. CFPAC-1 cells were treated with different concentrations of PDK1 inhibitors and their effects on cell viability (a, b) and anchorage independent growth (c, d) were assessed. Data are expressed as percentage of control cells treated with DMSO and are means ± SEM of n ≥ 3 independent experiments performed in duplicate. Statistical analysis was performed using GraphPad Prism version 6.0 and one-way ANOVA with Dunnett’s multiple comparisons test. *p < 0.05, **p < 0.01, ***p < 0.001, ****p < 0.0001 vs control. Figure S2. Effect of pharmacological PDK1 inhibition on PANC-1 cells. PANC-1 cells were treated with different concentrations of PDK1 inhibitors and their effects on cell viability (a, b) and anchorage independent growth (c, d) were assessed. Data are expressed as percentage of control cells treated with DMSO and are means ± SEM of n ≥ 3 independent experiments performed in duplicate. Statistical analysis was performed using GraphPad Prism version 6.0 and one-way ANOVA with Dunnett’s multiple comparisons test. *p < 0.05, **p < 0.01, ***p < 0.001, ****p < 0.0001 vs control. Figure S3. Representative images of 3D colonies of AsPC-1 and PANC-1 cells treated with PDK1 inhibitors. Images of AsPC-1 colonies treated with different concentrations of GSK2344470 (a) as well as PANC-1 colonies treated with MP7 (b) and GSK2344470 (c) were acquired using 4X magnification lens. (d) Images of the 6-well plates of PANC-1 colonies treated with GSK2344470 (left) and MP7 (right), as visualized by the ChemiDoc system (BioRad). Figure S4. Effect of pharmacologicalinhibition of PDK1 on HPAF-II cells anchorage–independent growth. HPAF-II cells were treated with the indicated concentrations of the PDK1 inhibitors GSK2334470, 2-O-Bn-InsP5 (a) and MP7 (b) and their effects on anchorage-independent growth were determined. Data are expressed as percentage of control cells treated with DMSO. Data in (a) are means ± SEM o [file 13046_2019_1191_MOESM2_ESM.zip › Emmanouilidi et al Addtional file 2 S4.tif]

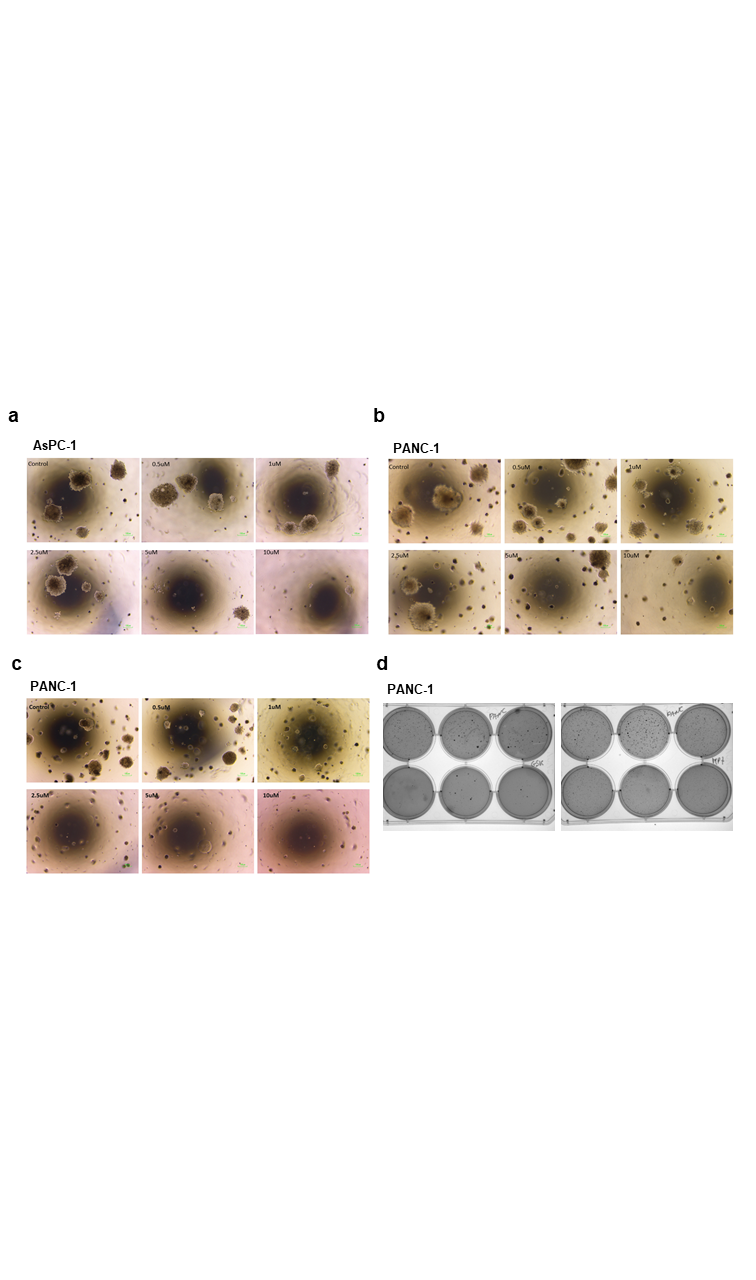

Supplement: Supplementary file 2 — Figure S1. Effect of pharmacological inhibition of PDK1 on CFPAC-1 cells. CFPAC-1 cells were treated with different concentrations of PDK1 inhibitors and their effects on cell viability (a, b) and anchorage independent growth (c, d) were assessed. Data are expressed as percentage of control cells treated with DMSO and are means ± SEM of n ≥ 3 independent experiments performed in duplicate. Statistical analysis was performed using GraphPad Prism version 6.0 and one-way ANOVA with Dunnett’s multiple comparisons test. *p < 0.05, **p < 0.01, ***p < 0.001, ****p < 0.0001 vs control. Figure S2. Effect of pharmacological PDK1 inhibition on PANC-1 cells. PANC-1 cells were treated with different concentrations of PDK1 inhibitors and their effects on cell viability (a, b) and anchorage independent growth (c, d) were assessed. Data are expressed as percentage of control cells treated with DMSO and are means ± SEM of n ≥ 3 independent experiments performed in duplicate. Statistical analysis was performed using GraphPad Prism version 6.0 and one-way ANOVA with Dunnett’s multiple comparisons test. *p < 0.05, **p < 0.01, ***p < 0.001, ****p < 0.0001 vs control. Figure S3. Representative images of 3D colonies of AsPC-1 and PANC-1 cells treated with PDK1 inhibitors. Images of AsPC-1 colonies treated with different concentrations of GSK2344470 (a) as well as PANC-1 colonies treated with MP7 (b) and GSK2344470 (c) were acquired using 4X magnification lens. (d) Images of the 6-well plates of PANC-1 colonies treated with GSK2344470 (left) and MP7 (right), as visualized by the ChemiDoc system (BioRad). Figure S4. Effect of pharmacologicalinhibition of PDK1 on HPAF-II cells anchorage–independent growth. HPAF-II cells were treated with the indicated concentrations of the PDK1 inhibitors GSK2334470, 2-O-Bn-InsP5 (a) and MP7 (b) and their effects on anchorage-independent growth were determined. Data are expressed as percentage of control cells treated with DMSO. Data in (a) are means ± SEM o [file 13046_2019_1191_MOESM2_ESM.zip › Emmanouilidi et al Addtional file 2 S3.tif]

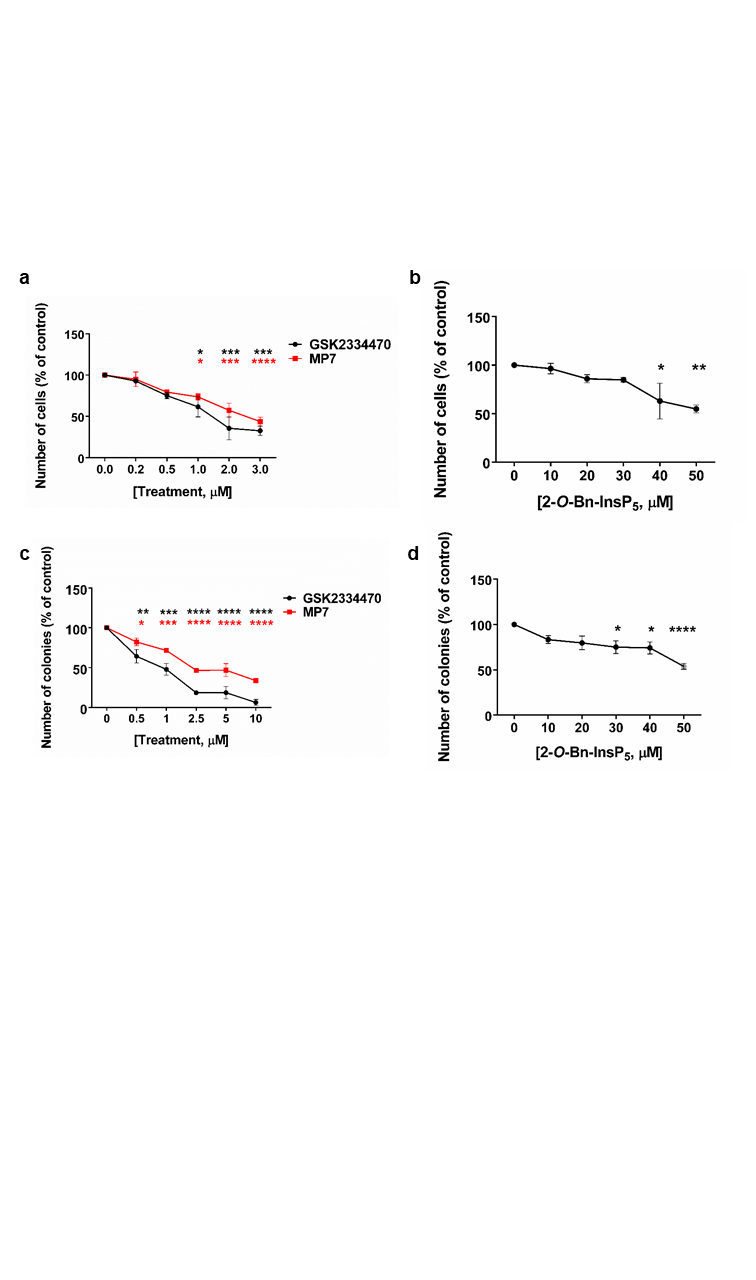

Supplement: Supplementary file 2 — Figure S1. Effect of pharmacological inhibition of PDK1 on CFPAC-1 cells. CFPAC-1 cells were treated with different concentrations of PDK1 inhibitors and their effects on cell viability (a, b) and anchorage independent growth (c, d) were assessed. Data are expressed as percentage of control cells treated with DMSO and are means ± SEM of n ≥ 3 independent experiments performed in duplicate. Statistical analysis was performed using GraphPad Prism version 6.0 and one-way ANOVA with Dunnett’s multiple comparisons test. *p < 0.05, **p < 0.01, ***p < 0.001, ****p < 0.0001 vs control. Figure S2. Effect of pharmacological PDK1 inhibition on PANC-1 cells. PANC-1 cells were treated with different concentrations of PDK1 inhibitors and their effects on cell viability (a, b) and anchorage independent growth (c, d) were assessed. Data are expressed as percentage of control cells treated with DMSO and are means ± SEM of n ≥ 3 independent experiments performed in duplicate. Statistical analysis was performed using GraphPad Prism version 6.0 and one-way ANOVA with Dunnett’s multiple comparisons test. *p < 0.05, **p < 0.01, ***p < 0.001, ****p < 0.0001 vs control. Figure S3. Representative images of 3D colonies of AsPC-1 and PANC-1 cells treated with PDK1 inhibitors. Images of AsPC-1 colonies treated with different concentrations of GSK2344470 (a) as well as PANC-1 colonies treated with MP7 (b) and GSK2344470 (c) were acquired using 4X magnification lens. (d) Images of the 6-well plates of PANC-1 colonies treated with GSK2344470 (left) and MP7 (right), as visualized by the ChemiDoc system (BioRad). Figure S4. Effect of pharmacologicalinhibition of PDK1 on HPAF-II cells anchorage–independent growth. HPAF-II cells were treated with the indicated concentrations of the PDK1 inhibitors GSK2334470, 2-O-Bn-InsP5 (a) and MP7 (b) and their effects on anchorage-independent growth were determined. Data are expressed as percentage of control cells treated with DMSO. Data in (a) are means ± SEM o [file 13046_2019_1191_MOESM2_ESM.zip › Emmanouilidi et al Addtional file 2 S2.tif]

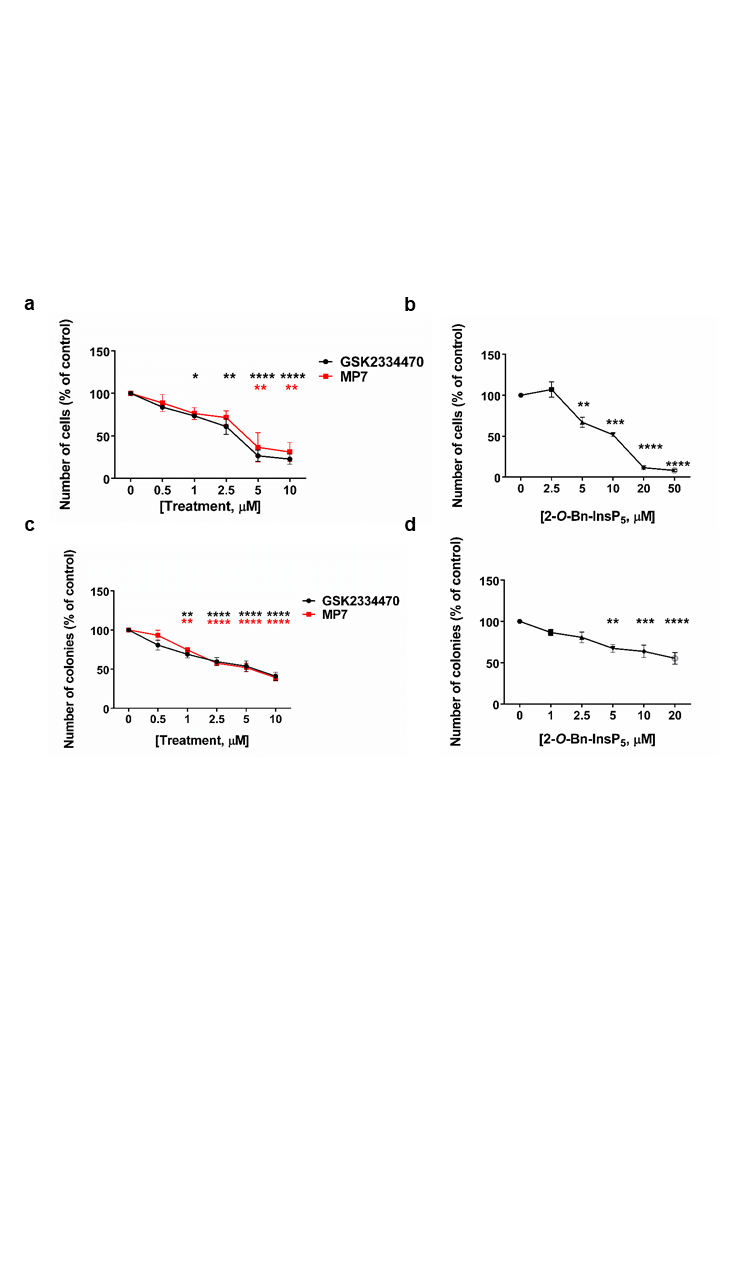

Supplement: Supplementary file 2 — Figure S1. Effect of pharmacological inhibition of PDK1 on CFPAC-1 cells. CFPAC-1 cells were treated with different concentrations of PDK1 inhibitors and their effects on cell viability (a, b) and anchorage independent growth (c, d) were assessed. Data are expressed as percentage of control cells treated with DMSO and are means ± SEM of n ≥ 3 independent experiments performed in duplicate. Statistical analysis was performed using GraphPad Prism version 6.0 and one-way ANOVA with Dunnett’s multiple comparisons test. *p < 0.05, **p < 0.01, ***p < 0.001, ****p < 0.0001 vs control. Figure S2. Effect of pharmacological PDK1 inhibition on PANC-1 cells. PANC-1 cells were treated with different concentrations of PDK1 inhibitors and their effects on cell viability (a, b) and anchorage independent growth (c, d) were assessed. Data are expressed as percentage of control cells treated with DMSO and are means ± SEM of n ≥ 3 independent experiments performed in duplicate. Statistical analysis was performed using GraphPad Prism version 6.0 and one-way ANOVA with Dunnett’s multiple comparisons test. *p < 0.05, **p < 0.01, ***p < 0.001, ****p < 0.0001 vs control. Figure S3. Representative images of 3D colonies of AsPC-1 and PANC-1 cells treated with PDK1 inhibitors. Images of AsPC-1 colonies treated with different concentrations of GSK2344470 (a) as well as PANC-1 colonies treated with MP7 (b) and GSK2344470 (c) were acquired using 4X magnification lens. (d) Images of the 6-well plates of PANC-1 colonies treated with GSK2344470 (left) and MP7 (right), as visualized by the ChemiDoc system (BioRad). Figure S4. Effect of pharmacologicalinhibition of PDK1 on HPAF-II cells anchorage–independent growth. HPAF-II cells were treated with the indicated concentrations of the PDK1 inhibitors GSK2334470, 2-O-Bn-InsP5 (a) and MP7 (b) and their effects on anchorage-independent growth were determined. Data are expressed as percentage of control cells treated with DMSO. Data in (a) are means ± SEM o [file 13046_2019_1191_MOESM2_ESM.zip › Emmanouilidi et al Addtional file 2 S1.tif]
